# Pre-operative MRI in evaluating pathologic complete response to neoadjuvant chemotherapy in patients with breast cancer: a study focused on influencing factors of baseline clinical-pathological and imaging features

**DOI:** 10.3389/fonc.2024.1366613

**Published:** 2024-05-17

**Authors:** Qilan Hu, Yiqi Hu, Huiyang Ai, Liming Xia, Rong Liu, Tao Ai

**Affiliations:** ^1^ Department of Radiology, Tongji Hospital, Huazhong University of Science and Technology, Wuhan, China; ^2^ Department of Ophthalmology, Tongji Hospital, Tongji Medical College, Huazhong University of Science and Technology, Wuhan, China

**Keywords:** pathologic complete response, neoadjuvant chemotherapy, radiologic complete response, magnetic resonance imaging, breast cancer

## Abstract

**Purpose:**

To investigate what pre-treatment clinical-pathological features and MRI characteristics influence the performance of breast MRI in assessing the pathologic complete response (pCR) of breast cancer patients to Neoadjuvant Chemotherapy (NAC).

**Methods:**

A total of 225 patients with pathologically-confirmed breast cancer who underwent pre- and post-NAC breast MRI between January 2020 and April 2023 were retrospectively analyzed. All patients were categorized into radiologic complete response (rCR) and non-rCR groups based on pre-operative MRI. Univariable and multivariable logistic regression were used to identify independent clinicopathological and imaging features associated with imaging-pathological discordance. The performance of pre-operative MRI for predicting pCR to NAC was assessed according to the baseline characteristics of the clinicopathological data and pre-NAC MRI. In addition, the discrepancy between the pre-operative MRI and post-operative pathological findings was further analyzed by a case-control approach.

**Results:**

Among 225 patients, 99 (44.0%) achieved pCR after NAC. MRI showed the overall sensitivity of 97.6%, specificity of 58.6%, accuracy of 80.4%, a positive predictive value (PPV) of 75.0%, and a negative predictive value (NPV) of 95.1% in identifying pCR. Of baseline features, presence of ductal carcinoma *in situ* (DCIS) (OR, 3.975 [95% CI: 1.448–10.908], *p* = 0.007), luminal B (OR, 5.076 [95% CI: 1.401–18.391], p = 0.013), HER2-enriched subtype (OR, 10.949 [95% CI: 3.262–36.747], *p* < 0.001), multifocal or multicentric lesions (OR, 2.467 [95% CI: 1.067–5.706], *p* = 0.035), segmental or regional distribution of NME (OR, 8.514 [95% CI: 1.049–69.098], *p* = 0.045) and rim enhancement of mass (OR, 4.261 [95% CI: 1.347–13.477], *p* = 0.014) were significantly associated with the discrepancy between MRI and pathology.

**Conclusion:**

Presence of DCIS, luminal B or HER2-enriched subtype, multicentric or multifocal lesions, segmental or regional distribution of NME and rim enhancement of mass may lead to a decrease in diagnostic accuracy of MRI in patients of breast cancer treated with NAC.

## Introduction

Based on the latest World Cancer Report 2020, an estimated 2.26 million new cases of breast cancer occurred worldwide (1). Breast cancer has surpassed lung cancer as the most prevalent malignant tumor in women, posing a grave threat to women’s health and quality of life (1). With neoadjuvant chemotherapy (NAC) becoming more available and effective for patients with locally advanced breast cancer, the primary objective of NAC has evolved from merely downstaging inoperable breast cancer to achieving pathological complete response (pCR). In previous studies, it has been demonstrated that patients who achieve pCR after NAC treatment show a significant improvement in both disease-free survival and overall survival rates (2–4).

Many researchers have attempted to explore whether imaging-guided pathological vacuum-assisted biopsy (VAB) could be a substitute for surgery in patients who achieved radiologic complete response (rCR) or near-rCR (5, 6). However, the rate of false negative (FN) in these trials ranged from 17.8% to 77.5%, which was not encouraging. The inability to accurately assess the residual tumor extent following NAC may lead to false-negative results, thereby compromising the adequacy of surgical approach and resection scope determined by clinicians. Consequently, this could result in an elevation of positive surgical margin rates and re-excision rates. Recent publications have indicated that the combined sensitivity of magnetic resonance imaging (MRI) in detecting the extent of residual disease ranges between 63% and 88%, with a specificity ranging from 54% to 91% (7). Breast magnetic resonance imaging (MRI), a non-invasive tool can provide quantitative information into microstructural changes in tumors with high heterogeneity during treatment, making it of great significance in therapeutic planning and evaluation. Moreover, MRI has been demonstrated to be the most sensitive tool in tumor response assessment among imaging techniques (5, 8). However, the application of MRI in predicting pCR currently falls short of clinical needs based on the available research findings. Despite the fact that MRI can provide a relatively accurate prediction of treatment response following NAC in most cases, there still exists discordances between MRI findings and post-operative pathological results (5, 9, 10). An increasing number of patients and physicians are eager for the non-invasive methods to accurately predict and diagnose pCR over the past decade. It is crucial to identify the features in MRI evaluations that contribute to the underestimation or overestimation of breast lesions. Few published studies investigated what tumor characteristics of initial MRI are associated with imaging-pathological discrepancy. This study, therefore, was to explore what pre-treatment clinical-pathological and MRI features influence the performance of breast MRI in assessing responses to NAC.

## Materials and methods

### Population selection

The institutional ethics review committee at our institution approved this study and the informed consent was obtained. From MRI database, 262 consecutive women with pathologically confirmed invasive breast cancer who underwent MRI scanning before and after NAC, followed by lumpectomy or mastectomy between February 2019 and April 2023 were retrospectively analyzed.

### MRI protocols

All breast MR studies were performed using the same high-field system (3.0T, Magnetom Skyra, Siemens Healthcare, Erlangen, Germany) with a dedicated bilateral 16-channel breast array coil. All Patients underwent MRI examinations both before and after NAC in a prone position. the protocol consisted of the following sequences:(a) a localizing acquisition, (b) an axial T2-weighted fast spin-echo sequence (TR/TE, 3700/101 ms; field of view, 320×320; matrix, 224×320; thickness of section, 4 mm; and acquisition time 2min:6sec), (c) a dynamic contrast-enhanced MRI (DCE-MRI) protocol based on the volume-interpolated breath-hold examination sequence (TWIST-VIBE) with parameters: TR/TE, 5.24/2.46 ms; flip angle,10°; field of view, 320×320; matrix, 182×320; thickness of section, 1.5mm; and acquisition time of 5min:57sec. It was conducted both before and after the administration of contrast materials, having a temporal resolution of 5.74 seconds and a consecutive acquisition of 60 phases. At the end of the third phase of scanning, the contrast agent (Omniscan, GE Healthcare, Milwaukee, WI) was intravenously injected via an automated high-pressure injector through the antecubital vein. The bolus injection dose was 0.1 mmol/kg of body weight, followed by a 20-ml saline flush with the same rate of 2.5 mL/sec.

### MRI image analysis

Assessment of breast MR images were performed according to the 5th edition Breast Imaging Reporting and Data System (BI-RADS^®^) lexicon (11), including amount of fibroglandular tissue (FGT; almost entirely fat or scattered fibroglandular tissue, heterogeneous fibroglandular tissue or extreme fibroglandular tissue), level of background parenchymal enhancement (BPE; minimal or mild, moderate or marked), type of time-intensity curves (TIC; persistent, plateau, washout) (12) and tumor morphological features. Type of lesions were classified into mass, non-mass enhancement (NME) or mass with NME. Lesions were categorized based on their distribution as single lesion, multifocal or multicentric lesion (13). Peritumoral edema defined as the water-like high-signal area surrounding the tumor on T2-weighted magnetic resonance imaging (T2WI). Water-like signal intensity on T2WI without enhancement on dynamic enhanced sequences was defined as intratumoral necrosis. The analysis of breast MRI findings before NAC was independently performed by one radiologist with 11 years of experience in breast MRI. For pre-operational MRI, the absence of enhancement in the area of the previous tumor bed on both early-phase and delayed-phase MR images was utilized as the assessment criterion of rCR, consistent with the majority of previously published articles (5, 7, 13). The analyses of breast MRI findings after NAC were independently performed by three radiologists who were blinded to the pathological findings, with 3 years, 6 years, and 11 years of experience in breast MRI, respectively. When the discordant case appeared, the consensus was reached through reevaluation and discussion.

### Clinical-pathologic data collection

Clinical and pathologic data were obtained from the clinical medical record system, including patients’ age at initial diagnosis, menopausal status, tumor subtype, WHO-grade, clinical T stage, chemotherapy protocol, histologic type, presence of ductal carcinoma *in situ* (DCIS), status of axillary lymph nodes and hormone receptors, and Ki-67 expression.

### Histopathologic analysis

The molecular subtypes of breast cancer was categorized according to the immunohistochemical data (14). The definition of hormone receptor (HR) positivity based on immunohistochemistry analysis was as follows: estrogen receptor (ER) and/or progesterone receptor (PR) positivity (≥ 10% tumor cell nuclei staining). HER2 was considered positive with an IHC score of 3+ or 2+ with gene amplification by fluorescence *in situ* hybridization technique in tumor analysis. Specimens for all cases prior to chemotherapy were classified as follows: luminal A (ER and/or PR positive and HER2 negative), luminal B (ER and/or PR positive, HER2 positive), HER2-enriched (ER and PR negative, HER2 positive), and triple-negative (ER and PR negative, HER2 negative). The corresponding pathological tumor response was assessed by utilizing the Miller and Payne grading system after surgical treatment (15). As previously depicted by Harada et al (16, 17), a diagnosis of pCR was provided when the components of invasive cancer cells in the breast dissolved, regardless of the presence of DCIS or axillary metastasis.

### Statistical analysis

Statistical analyses were performed using SPSS for Windows (version 26.0; SPSS, Inc, Chicago, IL, USA). The Shapiro-Wilk test was used to assess the distribution normality of the data. Subsequently, data were expressed as the mean ± standard deviation for continuous variables, and as percentages for categorical variables. The Student’s t-test was used to compare the continuous variables, and the Chi-square test for categorical variables among different groups. Univariable and multivariable logistic regression analyses were performed to determine if any factors were independently associated with imaging-pathologic discordance. All variables with *p* < 0.10 in the univariable analysis can enter into the multivariable analysis.

The diagnostic performance of MRI in differentiating pCR from residual cancer lesions including sensitivity, specificity, accuracy, positive predictive value (PPV), and negative predictive value (NPV) were calculated. For the sake of assessing MRI performance to detect patients who had achieved pCR after NAC, we regarded rCR as the “negative” result. If a case was diagnosed as rCR by breast MRI and pCR by the pathological analysis for post-operative pathological sections, a “true negative” (TN) result was considered. True positive (TP) was defined as non-rCR on MRI and non-pCR on pathology; FN was rCR on MRI but non-pCR on pathology; and false positive (FP) was non-rCR on MRI and pCR on pathology. When the FP or FN results occurred, we defined them as image-pathologic discordance. In contrast, either TP or TN results were considered image-pathologic concordance.

The variability among three observers for the MRI response evaluations was assessed by using intraclass correlation coefficient (ICC). Interpretation criteria of agreement was as follows: 0.00–0.20, poor; 0.21–0.40, fair; 0.41–0.60, moderate; 0.61–0.80, substantial; and 0.81–1.00, almost perfect. A p-value<0.05 was considered to be of significance in this setting.

## Results

### Patients and tumor characteristics

A summary of patients’ features displays based on responses in **Table 1**. The excluded patients included (1): patients were excluded since MRI had not been performed before or after NAC (n = 17) (2); patients with unavailability of pathological data (n = 6), patients with the poor quality of images (n = 5) (3); patients with chemotherapy treatment interruption due to toxic side effects or distant metastases (n = 3) (4); patients with loss to follow-up (n = 3) (5); patients did not receive definitive surgery after NAC (n = 3). As a result, a total of 225 patients with a mean age of 47.3 years (range: 20–74 years) were enrolled in this final cohort. Among the 225 patients, the overall pCR rate was 44.0% in our study. With regard to tumor subtypes, the vast majority was luminal A (32.4%), followed by HER2-enriched (24.4%), triple-negative (24%), and luminal B subtype (19.1%). Tumor subtype remained a significant predictor of pCR, with non-luminal A subtype demonstrating higher odds of pCR (*p* < 0.001). In terms of histological grading, patients with grade I-II showed a significantly lower rate of pCR compared to those with grade III (*p* = 0.004). The remaining 73 cases were not included in the analysis due to missing data. Other factors significantly correlated with pCR encompass menopausal status, the regimen of NAC, tumor subtype, expression of Ki-67, and the status of axillary lymph nodes (all *p* < 0.05). No significant association was observed between MRI characteristics and pCR.

**Table 1:** Baseline MRI and Clinical-Pathologic characteristics according to pathologic Response.

Characteristics (n=225)	pCR (n=99)	Residual cancer (n=126)	*p-*value
Age (mean ± SD, years)	48.8 ± 9.9	46.1 ± 11.9	0.067
Menopausal status			0.004*
Premenopausal	48 (48.5%)	78 (61.9%)	
Postmenopausal	51(51.5%)	48 (38.1%)	
WHO histologic grading			0.004*
I-II	23 (23.2%)	80 (63.5%)	
III	22 (22.2%)	27 (21.4%)	
Missing	54(54.5%)	19 (15.1%)	
Clinical T stage			0.793
T1–2	58 (58.6%)	76 (60.3%)	
T3–4	41 (41.4%)	50 (39.7%)	
NAC regimen			< 0.001*
Taxane-based	27 (27.3%)	55 (43.7%)	
Anthracycline-based	6 (6.1%)	27 (21.4%)	
HER2-targeted	43 (43.4%)	23 (18.3%)	
Other	23 (23.2%)	21 (16.7%)	
Histological type			0.713
IDC	95 (96.0%)	119 (94.4%)	
ILC	2 (2.0%)	2 (1.6%)	
Other	2 (2.0%)	5 (4.0%)	
Tumor subtype			< 0.001*
Luminal A	10 (10.1%)	63 (50.0%)	
Luminal B	25 (25.3%)	18 (14.3%)	
HER2-enriched	45 (45.5%)	10 (7.9%)	
Triple-negative	19 (19.2%)	35 (27.8%)	
Ki-67 expression			0.014*
Low (<14%)	7 (7.1%)	23 (18.3%)	
High (≥14%)	92 (92.9%)	103 (81.7%)	
Axillary lymph node status			0.009*
Negative	33 (33.3%)	23 (18.3%)	
Positive	66 (66.7%)	103 (81.7%)	
Presence of DCIS			0.200
Negative	85 (85.9%)	115 (91.3%)	
Positive	14 (14.1%)	11 (8.7%)	
Days to surgery			0.304
0–14 days	88 (88.9%)	106 (84.1%)	
>14 days	11 (11.1%)	20 (15.9%)	
lesion size (mm)			0.562
Mean age ± SD	50.1 ± 22.7	48.4 ± 19.1	
Median (range)	42 (15–135)	45 (15–110)	
FGT			0.579
a or b	21 (21.2%)	23 (18.3%)	
c or d	78 (78.8%)	103 (81.7%)	
Level of BPE			0.369
Minimal or Mild	67 (67.7%)	78 (61.9%)	
Moderate or Marked	32 (32.3%)	48 (38.1%)	
Peritumoral edema			0.302
Negative	45 (45.5%)	66 (52.4%)	
Positive	54 (54.5%)	60 (47.6%)	
Intratumoral necrosis			0.801
Negative	66 (66.7%)	86 (68.3%)	
Positive	33 (33.3%)	40 (31.7%)	
Tumor distribution			0.079
Single	58 (58.6%)	88 (69.8%)	
Multifocal or Multicentric	49 (41.4%)	47 (30.2%)	
Lesion type			0.098
Mass	47 (47.5%)	73 (57.9%)	
NME	28 (28.3%)	36 (28.6%)	
Mass with NME	24 (24.2%)	17 (13.5%)	
TIC types of lesions			0.115
Persistent	1 (1.0%)	6 (4.8%)	
Plateau	37 (37.4%)	56 (44.4%)	
Washout	61 (61.6%)	64 (50.8%)	

Data are expressed as percentages of patients and as mean ± SD.

pCR, complete pathological response; IDC, invasive ductal carcinoma; ILC, invasive lobular carcinoma; HER2, human epidermal growth factor receptor 2; FGT, fibroglandular tissue; BPE, background parenchymal enhancement; NME, Non-Mass Enhancement; TIC, time-intensity curves; DCIS, ductal carcinoma in situ.

*Represents a P-value less than 0.05.

### Univariable and multivariable analyses associated with MRI accuracy

On the univariable logistic regression analysis (**Table 2**), T3–4 stage (OR, 2.042 [95% CI: 1.049–3.973], *p* = 0.036), presence of DCIS (OR, 4.846 [95% CI: 2.028–11.578], *p* < 0.001), luminal B (odds ratio [OR], 5.930 [95% CI: 1.753–20.059], *p* = 0.004), HER2-enriched subtype (OR, 12.398 [95% CI: 3.959–38.827], *p* < 0.001), high Ki-67 expression(OR, 3.843 [95% CI: 0.880–16.792], *p* = 0.074), multifocal or multicentric lesions(OR, 2.175 [95% CI: 1.114–4.247], *p* = 0.023), NME (OR, 2.333 [95% CI: 1.067–5.104], *p* = 0.034), mass and NME (OR, 3.250 [95% CI: 1.387–7.617], *p* = 0.007), segmental or regional NME (OR, 8.514 [95% CI: 1.049–69.098], *p* = 0.045), and rim enhancement of mass (OR, 4.261 [95% CI: 1.347–13.477], *p* = 0.014) were associated with higher odds of imaging-pathological discordance.

**Table 2:** Univariable logistic regression analysis for characteristics associated with MRI accuracy.

Characteristics	Concordant (n=181)	Discordant (n=44)	Odds ratio (95% CI)	*p-*value
Age (mean ± SD, years)	47.2 ± 11.4	47.7 ± 10.4	1.005 (0.975–1.035)	0.757
Menopausal status				0.372
Premenopausal	104	22	reference	
Postmenopausal	77	22	1.351(0.698–2.614)	
WHO histologic grading				0.006*
I-II	91	12	reference	
III	40	9	1.706 (0.666–4.372)	0.266
Missing	50	23	NA	
Clinical T stage				0.036*
T1–2	114	20	reference	
T3–4	67	24	2.042 (1.049–3.973)	
NAC regimen				0.120
Taxane-based	68	14	reference	
Anthracycline-based	31	2	0.313 (0.067–1.464)	0.140
HER2-targeted	49	17	1.685 (0.759–3.739)	0.199
Other	33	11	1.619 (0.663–3.952)	0.290
Histological type				0.932
IDC	171	43	reference	
ILC	4	0	NA	
Other	6	1	0.663 (0.078–5.652)	0.707
Presence of DCIS				<0.001*
Negative	168	32	reference	
Positive	13	12	4.846 (2.028–11.578)	
Tumor subtype				< 0.001*
Luminal A	69	4	reference	
Luminal B	32	11	5.930 (1.753–20.059)	0.004*
HER2-enriched	32	23	12.398 (3.959–38.827)	< 0.001*
Triple-negative	48	6	2.156 (0.577–8.053)	0.253
Ki-67 Expression				0.074*
Low (<14%)	28	2	reference	
High (≥ 14%)	153	42	3.843 (0.880–16.792)	
Axillary lymph node status				0.427
Negative	43	13	reference	
Positive	138	31	0.743 (0.357–1.546)	
Days to surgery				0.605
0–14 days	155	39		
>14 days	26	5	0.764 (0.276–2.118)	
FGT				0.867
a or b	35	9	reference	
c or d	146	35	0.932 (0.411–2.117)	
Level of BPE				0.901
Minimal or Mild	117	28	reference	
Moderate or Marked	64	16	1.045 (0.526–2.074)	
Peritumoral edema				0.215
Negative	93	18	reference	
Positive	88	26	1.527 (0.783–2.977)	
Intratumoral necrosis				0.536
Negative	124	28	reference	
Positive	57	16	1.243 (0.624–2.478)	
Tumor distribution				0.023*
Single	124	22	reference	
Multifocal or Multicentric	57	22	2.175 (1.114–4.247)	
Lesion type				0.015*
Mass	105	15	reference	
NME	48	16	2.333 (1.067–5.104)	0.034
Mass with NME	28	13	3.250 (1.387–7.617)	0.007
Distribution of NME				0.078
Linear or Focal	15	1	reference	
Segmental or Regional	37	21	8.514 (1.049–69.098)	0.045*
Multiple regional or Diffuse	24	7	4.375 (0.488–39.184)	0.187
Shape of mass				NA
Round or oval	11	0	reference	
Irregular	122	28	NA	
Spiculated mass margin				0.395
Absent	110	25	reference	
Present	23	3	0.574 (0.160–2.062)	
Internal enhancement of mass				0.483
Homogenous	13	4	reference	
Heterogenous	120	24	1.538 (0.462–5.125)	
Rim enhancement of mass				0.014*
Absent	125	22	reference	
Present	8	6	4.261 (1.347–13.477)	
TIC types of lesions				0.641
Washout	97	28	reference	
Plateau	77	16	0.720 (0.364–1.425)	0.346
Persistent	7	0	NA	

Data are expressed as proportions or mean ± SD.

CI, confidence interval; IDC, invasive ductal carcinoma; ILC, invasive lobular carcinoma; DCIS, ductal carcinoma in situ; HER2, Human Epidermal Growth Factor Receptor 2; FGT, fibroglandular tissue; BPE, background parenchymal enhancement; NME, Non-Mass Enhancement; TIC, time-intensity curves.

NA, not applicable.

*Represents a P-value less than 0.1.

On the multivariable logistic regression analysis (**Table 3**), presence of DCIS (OR, 3.975 [95% CI: 1.448–10.908], *p* = 0.007), luminal B (odds ratio [OR], 5.076 [95% CI: 1.401–18.391], *p* = 0.013), HER2-enriched subtype (OR, 10.949 [95% CI: 3.262–36.747], *p* < 0.001), multifocal or multicentric lesions(OR, 2.467 [95% CI: 1.067–5.706], *p* = 0.035) were independently associated with higher odds of imaging-pathological discordance.

**Table 3:** Multivariable logistic regression analysis for characteristics associated with MRI accuracy.

Characteristics	Odds ratio (95% CI)	p-value
Clinical T stage		0.239
T1–2	reference	
T3–4	1.694 (0.705–4.074)	
Ki-67 Expression		0.162
Low (<14%)	reference	
High (≥ 14%)	3.313 (0.618–17.774)	
Presence of DCIS		0.007*
Negative	reference	
Positive	3.975 (1.448–10.908)	
Tumor subtype		< 0.001*
Luminal A	reference	
Luminal B	5.076 (1.401–18.391)	0.013*
HER2-enriched	10.949 (3.262–36.747)	< 0.001*
Triple-negative	1.769 (0.440–7.117)	0.422
Tumor distribution		0.035*
Single lesion	reference	
Multifocal or Multicentric	2.467 (1.067–5.706)	
Lesion type		0.798
Mass	reference	
NME	1.358 (0.491–3.759)	0.555
Mass with NME	0.973 (0.346–2.736)	0.959

Data are expressed as proportions or mean ± SD.

CI, confidence interval; DCIS, ductal carcinoma in situ; HER2, Human Epidermal Growth Factor Receptor 2; NME, Non-Mass Enhancement;

*Represents a P-value less than 0.05.

### Diagnostic performance of MRI according to varied groups

The diagnostic performance of MRI prediction in each subgroup was summarized in **Table 4**. Of 225 patients, 61 were diagnosed as rCR by MRI analysis, while 99 achieved pCR by post-operative pathological analyses. Among three observers, the ICC value was 0.910 (95% CI: 0.888–0.928) for predicting pCR, which suggested the agreement was almost perfect. According to the current findings of our study, the overall diagnostic sensitivity, specificity, accuracy, PPV, and NPV of MRI were 97.6%, 58.6%, 80.4%, 75.0%, and 95.1%, respectively. Regarding tumor subtypes, the accuracy for the luminal A subtype was 94.5%, the highest score among all the subtypes. Compared to tumors without combined DCIS, the accuracy for tumors combined with DCIS was also significantly lower (52.0% vs. 84.0%). In addition, MRI accurately predicted pCR in 124 of 146 patients with single lesion (84.9%), while 57 of 79 patients with multifocal or multicentric lesions (72.2%). For NME lesions, MRI yielded the highest accuracy of 93.8% in lesions with a linear or focal distribution (93.8%). For mass lesion, MRI showed the higher accuracy in lesions without rim enhancement (85.0%).Diagnostic performance for predicting pCR was found to vary depending on subgroup variables.

**Table 4:** Diagnostic performance of MRI for detecting residual disease based on varied subgroups.

	Sensitivity (%)	Specificity(%)	Accuracy (%)	PPV (%)	NPV (%)
All subtypes	97.6	58.6	80.4	75.0	95.1
Molecular subtype					
Luminal A (n=73)	98.4	70.0	94.5	95.4	87.5
Luminal B (n=43)	94.4	60.0	74.4	63.0	93.8
HER2-enriched (n=55)	100.0	48.9	58.2	30.3	100.0
Triple-negative (n=54)	97.1	73.7	88.9	87.2	93.3
Presence of DCIS					
Negative (n=200)	97.4	65.9	84.0	79.4	94.9
Positive (n=25)	100.0	14.3	52.0	47.8	100.0
Tumor distribution					
Single (n=146)	97.7	65.5	84.9	81.1	95.0
Multifocal or Multicentric (n=79)	97.4	48.8	72.2	63.8	95.2
Distribution of NME (n=105)					
Linear or Focal (n=16)	87.5	100.0	93.8	100.0	88.9
Segmental or Regional (n=58)	96.0	39.4	63.8	54.5	92.9
Multiple regional or Diffuse (n=31)	100.0	36.4	77.4	74.1	100.0
Rim enhancement of mass (n=161)					
Absent (n=147)	97.6	67.7	85.0	80.6	95.5
Present (n=14)	100.0	33.3	57.1	45.5	100.0

PPV, Positive Predictive Value; NPV, Negative Predictive Value; HER2, Human Epidermal Growth Factor Receptor 2; NME, Non-Mass Enhancement; DCIS, Ductal Carcinoma In Situ.

### Analysis of cases with imaging-pathological discrepancy

**Figure 1:**
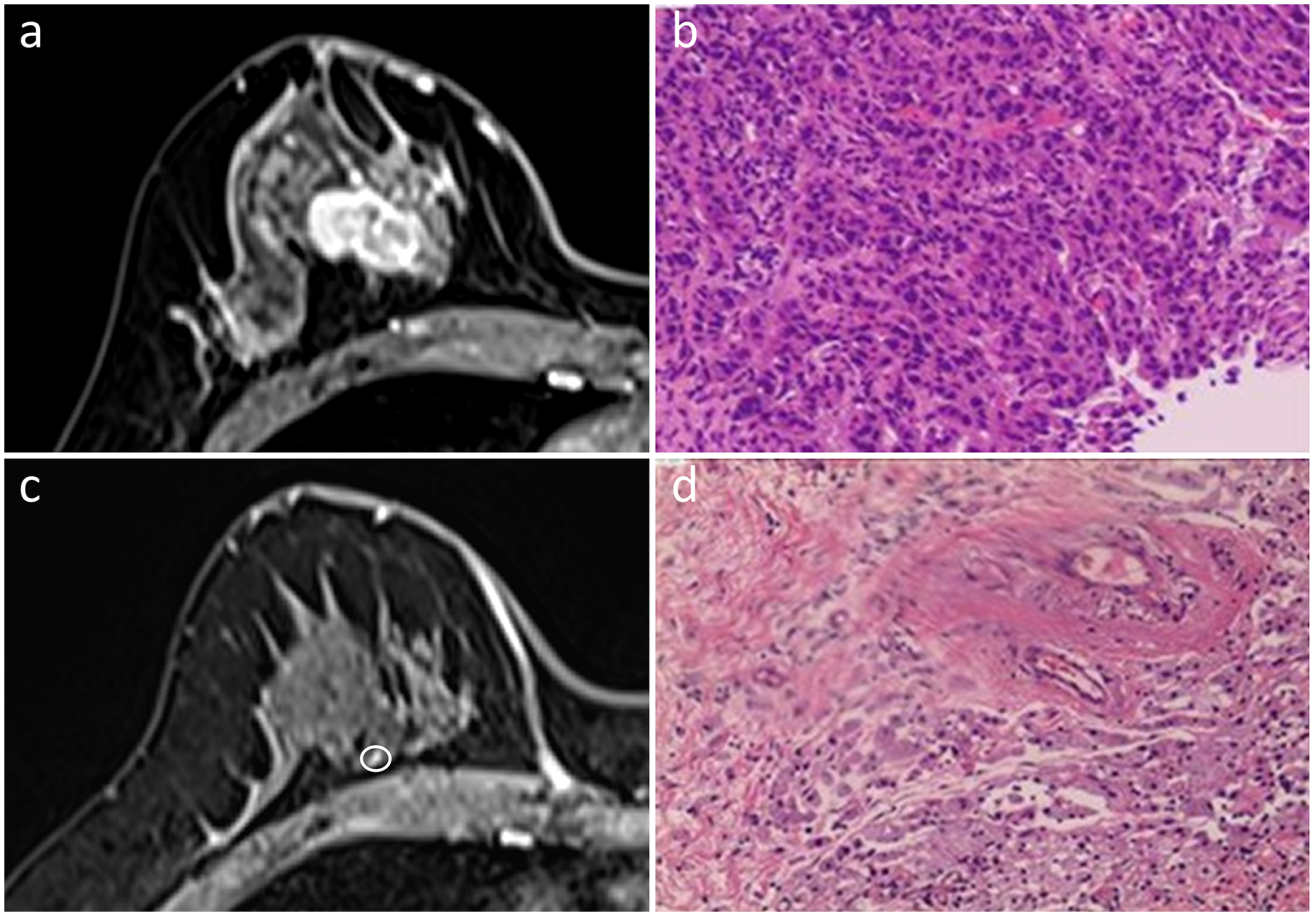
In the initial dynamic contrast-enhanced axial MR images **(A)**, a 40-year-old woman presented with a single irregular enhancing mass in the right breast. She was diagnosed with the triple-negative subtype of invasive ductal carcinoma **(B)**. An enhancing nodularity of 0.3 cm in size (circled) was detected on the MRI post-NAC **(C)**. While no residual invasive cancer cells were found, extensive fibrosis was observed in the interstitial space, indicative of pCR **(D)**. Hematoxylin and eosin staining was performed at a low magnification (×4, **B, D**).

**Figure 2:**
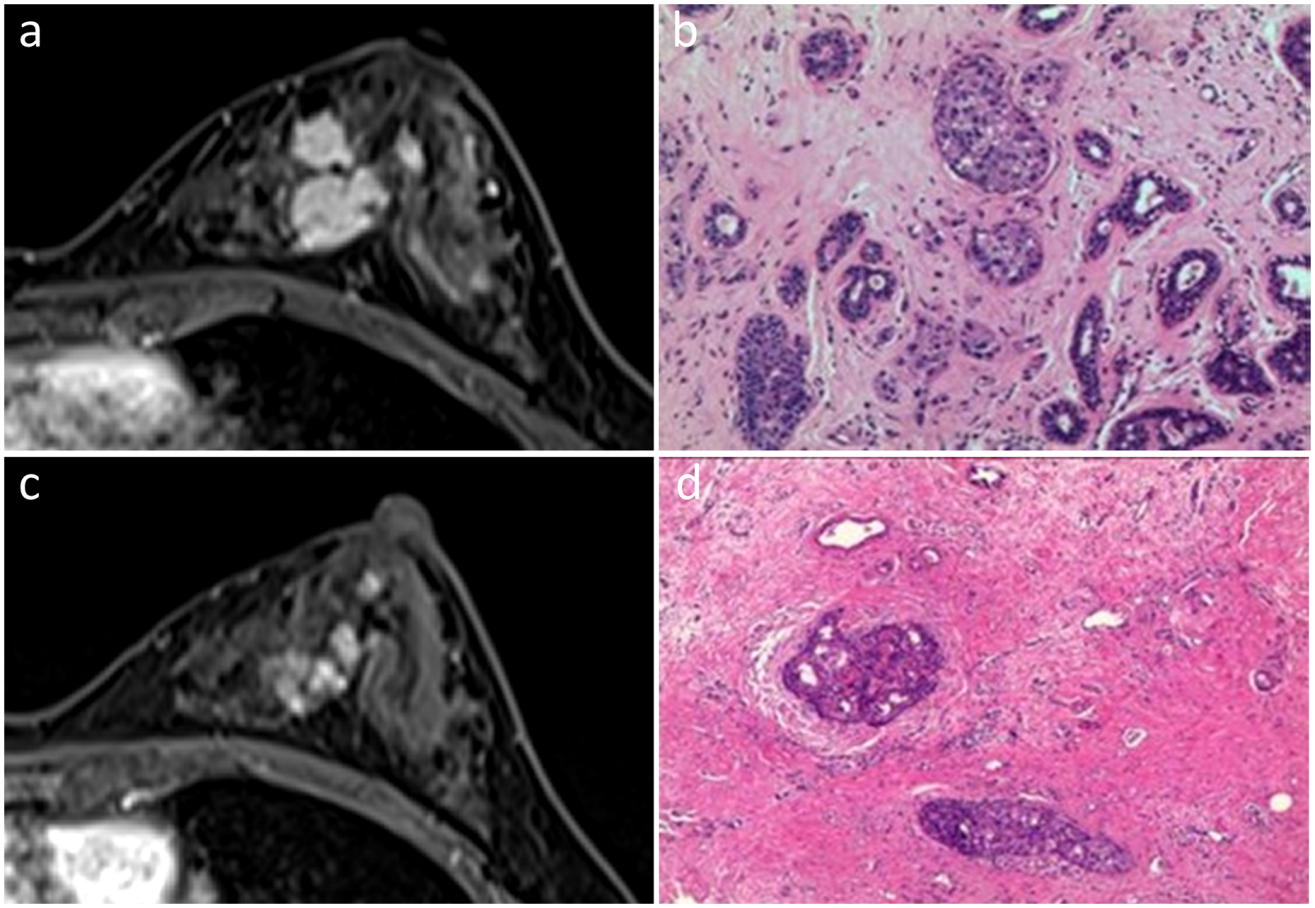
In the initial dynamic contrast-enhanced axial MR images **(A)**, a 45-year-old woman displayed multiple irregular enhancing masses with partial integration in the left breast. She was diagnosed with the Luminal A subtype of invasive ductal carcinoma **(B)**. Post-NAC MR images demonstrated regional NME, suggestive of non-rCR **(C)**. Pathological examination revealed pCR with residual DCIS **(D)**. Hematoxylin and eosin staining was performed at a low magnification (×10, **B, D**).

**Figure 3:**
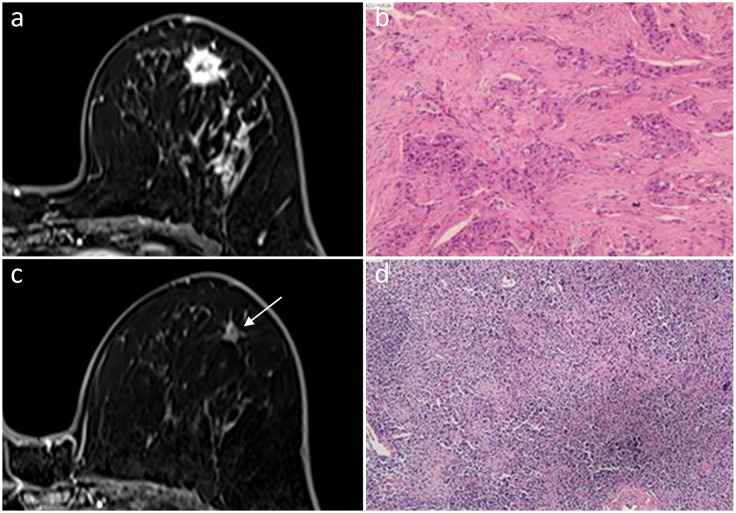
In a 56-year-old woman, an irregular mass with ring enhancement was observed in the left breast on initial dynamic contrast-enhanced axial MR images **(A)**. She was diagnosed with HER2-positive subtype invasive ductal carcinoma **(B)**. A 0.8-cm ring-enhanced lesion (arrow) was detected by MRI after NAC **(C)**. Fibrous tissue hyperplasia, accompanied by small vascular hyperplasia in the necrotic tumor bed, was observed, indicating pCR **(D)**. Hematoxylin and eosin staining is shown at low magnification (×4, **B, D**).

Among the 164 cases diagnosed with non-rCR, 41 cases (25.0%)were pathologically confirmed as pCR. Of these, 17 cases showed a single small nodularity of enhancement on DCE-MRI sequences (**Figure 1**). Extensive interstitial fibrosis and residual DCIS were found in 14 cases and 3 cases, respectively. NME lesions were observed in 19 cases, with low, medium, or high-grade DCIS detected in 12 cases (**Figure 2**). Interstitial changes induced by chemotherapy drugs were observed in the other 7 NME cases. 3 cases presented with an irregular mass showing mild ring enhancement. Pathology revealed necrosis of the tumor bed surrounded by fibrous connective tissue with proliferating new blood vessels (**Figure 3**). The remaining 2 cases displayed multiple small nodularities on MRI and extensive interstitial fibrosis or DCIS in the tumor area, as indicated by pathology.

**Figure 4:**
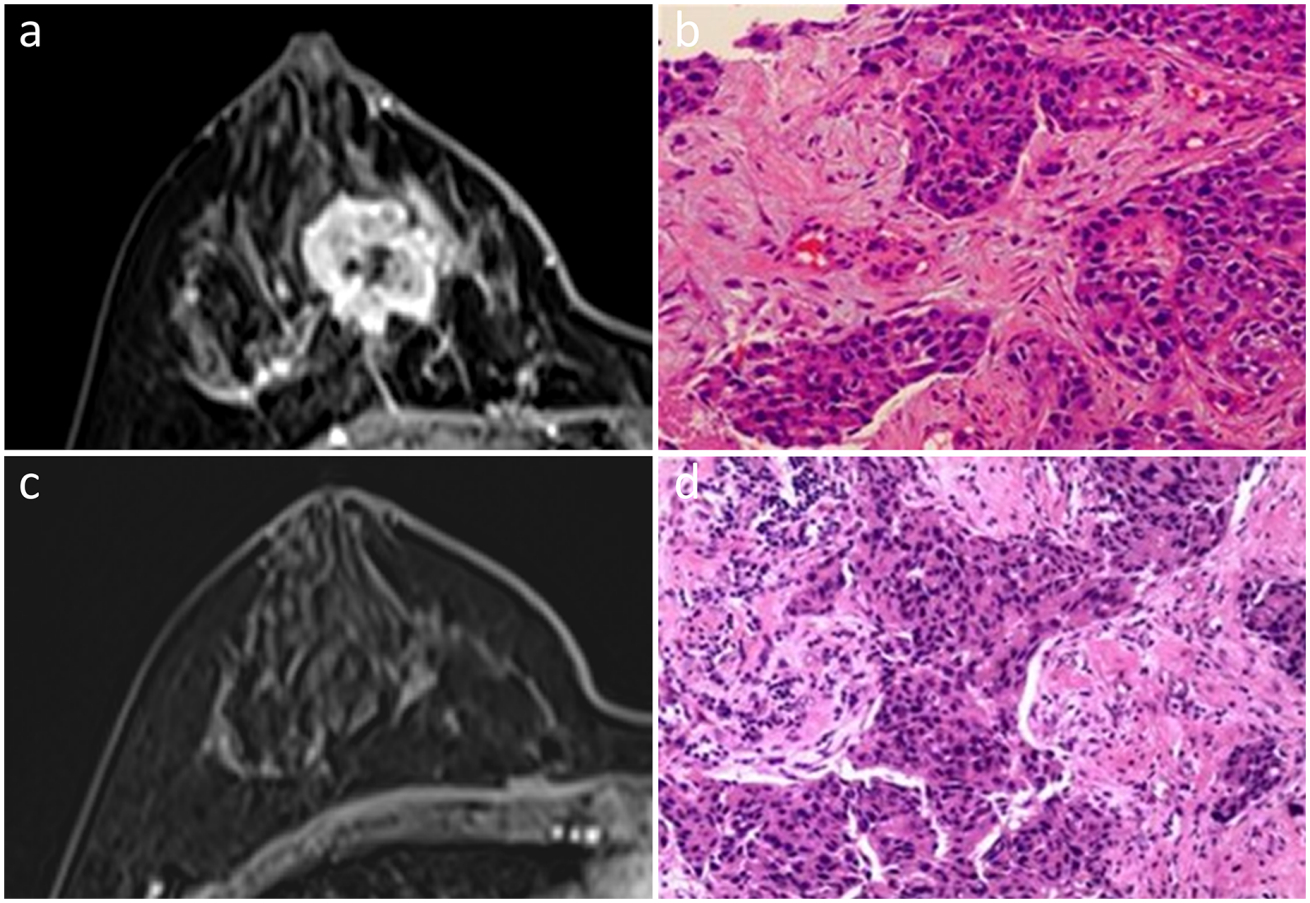
In a 47-year-old woman, an irregular mass with heterogeneous enhancement was observed in the right breast on initial dynamic contrast-enhanced axial MR images **(A)**. She was diagnosed with Luminal B subtype invasive ductal carcinoma **(B)**. No enhancement was found by MRI after NAC **(C)**. Scattered subtle invasive nests of residual cancer cells, combined with DCIS in the tumor bed, were observed upon pathological examination, indicating non-pCR **(D)**. Hematoxylin and eosin staining is shown at low magnification (×20, **B, D**).

Among the 61 cases diagnosed with rCR, 3 cases (5%) were pathologically confirmed not to have achieved pCR. These three cases were assessed as rCR on MRI after completing NAC. Of these, two were evaluated as G3 in the post-NAC surgical pathology, and the remaining one was assessed as G4. The former case showed only scattered small clusters of residual cancer cells with cytoplasmic consolidation and eosinophilic changes in post-operative pathology (**Figure 4**). In contrast, the latter displayed vacuolation of tumor cells (**Table 5**).

**Table 5:** Analysis of discrepancies between preoperative MR imaging and postoperative pathological findings.

MRI evaluation	MR Imaging findings	Largest lesion size	Pathologic findings	Tumor subtype	M&P grading
Non-rCR (n=41)	Single nodular enhancing foci (n=17)	2–5 mm	Interstitial fibrosis (n=14)	Luminal B (n=4)	G5
				HER2-enriched (n=9)	G5
				Triple-negative (n=1)	G5
			DCIS (n=3)	Luminal B(n=1)	G5
				HER2-enriched (n=1)	G5
				Triple-negative (n=1)	G5
	Irregular mass with mild ring enhancement (n=3)	10–20 mm	Necrosis of tumor bed surrounded by fibrous connective tissue (n=3)	Luminal B (n=1)	G5
				HER2-enriched (n=1)	G5
				Triple-negative (n=1)	G5
	Multiple nodular enhancing foci (n=2)	3, 4, 6 mm	DCIS (n=1)	HER2-enriched (n=1)	G5
		4, 5mm	Interstitial fibrosis (n=1)	HER2-enriched (n=1)	G5
	Non-Mass-Like enhancement (n=19)	4–42 mm	DCIS (n=12)	Luminal A (n=3)	G5
				Luminal B (n=3)	G5
				HER2-enriched (n=5)	G5
				Triple-negative (n=1)	G5
		7–43 mm	Interstitial fibrosis (n=7)	HER2-enriched (n=5)	G5
				Triple-negative (n=1)	G5
				Luminal B (n=1)	G5
rCR (n=3)	No enhancement		Scattered distribution of a few invasive cancer cells (n=2)	Luminal B (n=1)	G4
				Luminal A (n=1)	G3
			Vacuolization of partial tumor cells (n=1)	Triple-negative (n=1)	G3

rCR, radiologic Complete Response; HER-2, Human Epidermal Growth Factor Receptor 2; NME, Non-Mass Enhancement; DCIS, Ductal Carcinoma in Situ.

## Discussion

In our study, we evaluated the diagnostic performance of MRI in detecting pCR following NAC and investigated the factors correlated with discrepancies between imaging and pathological results. Clarifying the factors behind imaging-pathological discrepancies is essential for the accurate prediction of pCR using breast MRI. It facilitates a better interpretation of breast MRI in patients with specific clinical-pathological or imaging characteristics, potentially aiding in further tailored treatment planning post-NAC. Our study indicated that certain tumor features, including multifocal or multicentric lesions, segmental or regional NME distribution and rim enhancement of mass, were associated with higher likelihood of imaging-pathological discordance in post-NAC patients. Additionally, the presence of DCIS was associated with a greater probability of MRI overestimation or underestimation. Furthermore, tumor subtype remained a significant predictive factor of imaging-pathological discordance.

Our results showed that pre-operative MRI exhibited an accuracy of 80% in predicting pCR, which was in line with previous studies (18, 19). Nevertheless, the outcomes revealed 41 FP cases (93.2%) and 3 FN cases (6.8%). For FN cases, several studies (20–22) pointed out that MRI has a limited ability to detect and identify minimal residual disease after NAC (22). Scattered microscopic remnant cancer after NAC could result in misinterpretation as rCR by MRI. The increasing use of anti-angiogenic drugs in breast cancer patients may lead to decreased enhancement and poor visibility of lesions on DCE-MRI. This challenge is currently beyond the resolution of traditional breast MRI, but functional imaging and radiomic analysis hold promise for future solutions.

Among 41 FP cases, residual DCIS was observed in 16 cases. In our study, the residual DCIS component after NAC was referred as pCR according to the Miller-Payne histological grading system. In practice, the diagnostic accuracy of MRI varies depending on different pCR definitions in practical application (23). FP rate would be evidently reduced if we consider pCR as the complete absence of any invasive cancer or DCIS, as some authors suggested in several published studies (7, 24). DCIS refers to a non-invasive malignant tumor confined within the mammary duct, without breaking through the basal membrane. Treatments for DCIS primarily involves breast-conserving resection, eliminating the need for axillary or sentinel lymph node dissection, and often leads to favorable prognosis. Conversely, patients with invasive breast cancer generally have worse prognosis with increased risk of recurrence and metastasis as tumor size enlarges, the number of lymph node metastases increases, or distant metastasis occurs. These patients often require radical mastectomy combined with axillary or sentinel lymph node dissection. Subsequently, a comprehensive approach including radiotherapy, chemotherapy, endocrine therapy, and anti-HER2 targeted therapy is administered based on individual factors such as pathological molecular subtype and age. It is of great importance to make accurate differentiation between invasive breast cancer and DCIS by MRI for clinicians to determine tailored treatment strategies. In our study, MRI performed better in invasive tumors not combined with the DCIS group. The specificity in the subgroup with combined DCIS was 14.3%, yielding the lowest score among all the subgroups. Among 25 cases pathologically confirmed breast cancers combined with DCIS before NAC, 12 cases showed imaging-pathological discordance. Furthermore, 7 of these 12 cases exhibited residual DCIS according to post-operative pathology, which is often overestimated as residual cancer by MRI. It is extremely challenging to distinguish DCIS from invasive cancer on MRI. We hypothesize that the high rate of DCIS residual may be due to its lack of sensitivity to chemotherapeutic agents, as 11 out of 25 patients with DCIS persisted in the post-operative pathology.

We found that the tumor subtype remained a key contributing factors of MRI performance, which is consistent with previous studies (19, 25). It is interesting to note, however, our results suggested that the odds of imaging-pathological discordance in luminal B and HER2-enriched tumors was about 6 and 12 times higher than in luminal A subtype, respectively. Some authors proposed that MRI accuracy was higher in HER2-positive and triple-negative breast cancer and less in luminal breast cancer (9, 19, 26), whereas others proposed that MRI accuracy significantly decreased in patients treated with HER2-targeted agents, resulting in an overestimation of the extent of the residual lesions due to the increase in neovascular permeability (27), which may lead to overtreatment in this subtype. A consensus has not yet been reached at present on this issue. The main reason was probable that our cohort had a different composition of molecular subtypes compared to previous studies. In contrast to HER2-enriched subtype, the PPV and NPV for the luminal A subtype was 95.4% and 87.5%, respectively. This contributes to achieving the highest score of accuracy among all the subtypes, indicating that MRI is a valuable tool to evaluate response to chemotherapy for this subtype.

Our findings also revealed that lesion distribution at initial MRI affected MRI evaluation. To our knowledge, no prior study has reported that a marked decrease in MRI accuracy in cases with multifocal or multicentric lesions. Earlier studies had established a link between multicentric lesions visible in pre-NAC MRI and FN results, where post-NAC MRI indicated rCR (13, 24). However, we did not focus on patients who had a rCR on MRI. Moreover, most of the imaging-pathologic discordant cases were FP diagnoses in our study, with a rate as high as 93.2% (41/44). Changes induced by chemotherapy, such as fibrosis, inflammation, or granulation tissue, can mimic residual cancer in the original tumor bed (21), and this is particularly prevalent in cases with multifocal or multicentric lesions. In contrast to other similar studies (19, 28), our study delved deeper into the role of NME distribution in the response evaluation after NAC. In our study, MRI was less accurate in predicting pCR for segmental and regional NME. Several previous studies (29, 30) suggested that MRI generally overestimated nonfocal lesions, which were similar to the distribution of NME in our study. This may account for the finding in our study aptly. Studies with larger sample size are needed to validate our conclusions. For mass lesions, univariable analysis confirmed the influence of rim enhancement at pretreatment MRI on the performance of MRI after NAC. A previous study suggested that rim enhancement appeared to be associated with high Ki-67 expression (31), which had been demonstrated to be independently associated with pCR by Kim et al.

There are several limitations to our study worth mentioning. First, this was a single-center retrospective analysis conducted with a single machine and small sample. Second, other characteristics that might influence MRI accuracy, for instance, the shrinkage pattern and the apparent diffusion coefficient (ADC) values of tumor pre-NAC were not evaluated. Future studies are necessary to understand how to mitigate these factors and apply tailored interpretations when assessing pathological responses post-NAC. Additionally, the varying interval between MRI before operation and Surgical excision might be a minor limitation of this study, which was likely to have an impact on the reliability of the results. It is worth mentioning that the majority (86.2%, 194/225) of patients underwent breast MRI within 2 weeks prior to surgery.

In conclusion, presence of DCIS, the luminal B or HER2-enriched subtype, multifocal or multicentric lesions, segmental or regional NME and rim enhancement of mass were independently associated with decreased MRI accuracy. Hence, a post-NAC MRI should be analyzed cautiously and comprehensively because its diagnostic performance significantly depends on certain baseline clinical-pathological and imaging features.

## Data availability statement

The original contributions presented in the study are included in the article/supplementary material. Further inquiries can be directed to the corresponding authors.

## Ethics statement

The studies involving humans were approved by Tongji Hospital, Huazhong University of Science and Technology. The studies were conducted in accordance with the local legislation and institutional requirements. The participants provided their written informed consent to participate in this study.

## Author contributions

QH: Conceptualization, Writing – original draft. YH: Methodology, Writing – original draft. HA: Formal analysis, Writing – review & editing. LX: Writing – review & editing. RL: Supervision, Writing – review & editing. TA: Supervision, Writing – review & editing.

## References

[1] Sung H Ferlay J Siegel RL Laversanne M Soerjomataram I Jemal A. Global cancer statistics 2020: GLOBOCAN estimates of incidence and mortality worldwide for 36 cancers in 185 countries. CA Cancer J Clin. (2021) 71:209–49. doi: 10.3322/caac.21660 33538338

[2] Cortazar P Zhang L Untch M Mehta K Costantino JP Wolmark N. Pathological complete response and long-term clinical benefit in breast cancer: the CTNeoBC pooled analysis. Lancet. (2014) 384:164–72. doi: 10.1016/S0140-6736(13)62422-8 24529560

[3] Liedtke C Mazouni C Hess KR Andre F Tordai A Mejia JA. Response to neoadjuvant therapy and long-term survival in patients with triple-negative breast cancer. J Clin Oncol. (2008) 26:1275–81. doi: 10.1200/JCO.2007.14.4147 18250347

[4] King TA Morrow M. Surgical issues in patients with breast cancer receiving neoadjuvant chemotherapy. Nat Rev Clin Oncol. (2015) 12:335–43. doi: 10.1038/nrclinonc.2015.63 25850554

[5] Gampenrieder SP Peer A Weismann C Meissnitzer M Rinnerthaler G Webhofer J. Radiologic complete response (rCR) in contrast-enhanced magnetic resonance imaging (CE-MRI) after neoadjuvant chemotherapy for early breast cancer predicts recurrence-free survival but not pathologic complete response (pCR). Breast Cancer Res. (2019) 21:19. doi: 10.1186/s13058-018-1091-y 30704493 PMC6357474

[6] Gourd K. San antonio breast cancer symposium 2019. Lancet Oncol. (2020) 21:28. doi: 10.1016/S1470-2045(19)30830-7 31866168

[7] Santamaria G Bargallo X Fernandez PL Farrus B Caparros X Velasco M. Neoadjuvant systemic therapy in breast cancer: association of contrast-enhanced MR imaging findings, diffusion-weighted imaging findings, and tumor subtype with tumor response. Radiology. (2017) 283:663–72. doi: 10.1148/radiol.2016160176 27875106

[8] Marinovich ML Macaskill P Irwig L Sardanelli F Mamounas E von Minckwitz G. Agreement between MRI and pathologic breast tumor size after neoadjuvant chemotherapy, and comparison with alternative tests: individual patient data meta-analysis. BMC Cancer. (2015) 15:662. doi: 10.1186/s12885-015-1664-4 26449630 PMC4599727

[9] Kim J Han BK Ko EY Ko ES Choi JS Park KW. Prediction of pathologic complete response on MRI in patients with breast cancer receiving neoadjuvant chemotherapy according to molecular subtypes. Eur Radiol. (2022) 32:4056–66. doi: 10.1007/s00330-021-08461-0 34989844

[10] Sutton EJ Braunstein LZ El-Tamer MB Brogi E Hughes M Bryce Y. Accuracy of magnetic resonance imaging-guided biopsy to verify breast cancer pathologic complete response after neoadjuvant chemotherapy: A nonrandomized controlled trial. JAMA Netw Open. (2021) 4:e2034045. doi: 10.1001/jamanetworkopen.2020.34045 33449096 PMC7811182

[11] Radiology ACo D'Orsi CJ Sickles EA Mendelson EB Morris EA. ACR BI-RADS atlas: breast imaging reporting and data system; mammography, ultrasound, magnetic resonance imaging, follow-up and outcome monitoring, data dictionary. Reston, VA: ACR, American College of Radiology (2013).

[12] Kuhl CK Mielcareck P Klaschik S Leutner C Wardelmann E Gieseke J. Dynamic breast MR imaging: are signal intensity time course data useful for differential diagnosis of enhancing lesions? Radiology. (1999) 211:101–10. doi: 10.1148/radiology.211.1.r99ap38101 10189459

[13] Thompson BM Chala LF Shimizu C Mano MS Filassi JR Geyer FC. Pre-treatment MRI tumor features and post-treatment mammographic findings: may they contribute to refining the prediction of pathologic complete response in post-neoadjuvant breast cancer patients with radiologic complete response on MRI? Eur Radiol. (2022) 32:1663–75. doi: 10.1007/s00330-021-08290-1 34716780

[14] Hammond ME Hayes DF Dowsett M Allred DC Hagerty KL Badve S. American Society of Clinical Oncology/College of American Pathologists guideline recommendations for immunohistochemical testing of estrogen and progesterone receptors in breast cancer (unabridged version). Arch Pathol Lab Med. (2010) 134:e48–72. doi: 10.5858/134.7.e48 20586616

[15] Ogston KN Miller ID Payne S Hutcheon AW Sarkar TK Smith I. A new histological grading system to assess response of breast cancers to primary chemotherapy: prognostic significance and survival. Breast. (2003) 12:320–7. doi: 10.1016/s0960-9776(03)00106-1 14659147

[16] Harada TL Uematsu T Nakashima K Sugino T Nishimura S Takahashi K. Is the presence of edema and necrosis on T2WI pretreatment breast MRI the key to predict pCR of triple negative breast cancer? Eur Radiol. (2020) 30:3363–70. doi: 10.1007/s00330-020-06662-7 32062698

[17] Bouzon A Iglesias A Acea B Mosquera C Santiago P Mosquera J. Evaluation of MRI accuracy after primary systemic therapy in breast cancer patients considering tumor biology: optimizing the surgical planning. Radiol Oncol. (2019) 53:171–7. doi: 10.2478/raon-2019-0023 PMC6572491 31104001

[18] Eun NL Gweon HM Son EJ Youk JH Kim JA. Pretreatment MRI features associated with diagnostic accuracy of post-treatment MRI after neoadjuvant chemotherapy. Clin Radiol. (2018) 73:676 e9– e14. doi: 10.1016/j.crad.2018.02.008 29567270

[19] Negrao EMS Souza JA Marques EF Bitencourt AGV. Breast cancer phenotype influences MRI response evaluation after neoadjuvant chemotherapy. Eur J Radiol. (2019) 120:108701. doi: 10.1016/j.ejrad.2019.108701 31610321

[20] Chen JH Bahri S Mehta RS Kuzucan A Yu HJ Carpenter PM. Breast cancer: evaluation of response to neoadjuvant chemotherapy with 3.0-T MR imaging. Radiology. (2011) 261:735–43. doi: 10.1148/radiol.11110814 PMC3219909 21878615

[21] Wasser K Sinn HP Fink C Klein SK Junkermann H Ludemann HP. Accuracy of tumor size measurement in breast cancer using MRI is influenced by histological regression induced by neoadjuvant chemotherapy. Eur Radiol. (2003) 13:1213–23. doi: 10.1007/s00330-002-1730-6 12764635

[22] Price ER Wong J Mukhtar R Hylton N Esserman LJ. How to use magnetic resonance imaging following neoadjuvant chemotherapy in locally advanced breast cancer. World J Clin cases. (2015) 3:607–13. doi: 10.12998/wjcc.v3.i7.607 PMC4517335 26244152

[23] Marinovich ML Macaskill P Irwig L Sardanelli F von Minckwitz G Mamounas E. Meta-analysis of agreement between MRI and pathologic breast tumour size after neoadjuvant chemotherapy. Br J Cancer. (2013) 109:1528–36. doi: 10.1038/bjc.2013.473 PMC3776985 23963140

[24] Choi WJ Kim HH Cha JH Shin HJ Chae EY Yoon GY. Complete response on MR imaging after neoadjuvant chemotherapy in breast cancer patients: Factors of radiologic-pathologic discordance. Eur J Radiol. (2019) 118:114–21. doi: 10.1016/j.ejrad.2019.06.017 31439230

[25] Kim SY Cho N Park IA Kwon BR Shin SU Kim SY. Dynamic contrast-enhanced breast MRI for evaluating residual tumor size after neoadjuvant chemotherapy. Radiology. (2018) 289:327–34. doi: 10.1148/radiol.2018172868 30152744

[26] Murphy C Mukaro V Tobler R Asher R Gibbs E West L. Evaluating the role of magnetic resonance imaging post-neoadjuvant therapy for breast cancer in the NEONAB trial. Intern Med J. (2018) 48:699–705. doi: 10.1111/imj.13617 28869790

[27] Moon HG Han W Ahn SK Cho N Moon WK Im SA. Breast cancer molecular phenotype and the use of HER2-targeted agents influence the accuracy of breast MRI after neoadjuvant chemotherapy. Ann Surg. (2013) 257:133–7. doi: 10.1097/SLA.0b013e3182686bd9 22968080

[28] Chen JH Bahri S Mehta RS Carpenter PM McLaren CE Chen WP. Impact of factors affecting the residual tumor size diagnosed by MRI following neoadjuvant chemotherapy in comparison to pathology. J Surg Oncol. (2014) 109:158–67. doi: 10.1002/jso.23470 PMC4005994 24166728

[29] Partridge SC Gibbs JE Lu Y Esserman LJ Sudilovsky D Hylton NM. Accuracy of MR imaging for revealing residual breast cancer in patients who have undergone neoadjuvant chemotherapy. AJR Am J Roentgenol. (2002) 179:1193–9. doi: 10.2214/ajr.179.5.1791193 12388497

[30] Ko ES Han BK Kim RB Ko EY Shin JH Hahn SY. Analysis of factors that influence the accuracy of magnetic resonance imaging for predicting response after neoadjuvant chemotherapy in locally advanced breast cancer. Ann Surg Oncol. (2013) 20:2562–8. doi: 10.1245/s10434-013-2925-6 23463090

[31] Song SE Shin SU Moon HG Ryu HS Kim K Moon WK. MR imaging features associated with distant metastasis-free survival of patients with invasive breast cancer: a case-control study. Breast Cancer Res Treat. (2017) 162:559–69. doi: 10.1007/s10549-017-4143-6 28185146

